# Identification of key contributors in complex population structures

**DOI:** 10.1371/journal.pone.0177638

**Published:** 2017-05-16

**Authors:** Markus Neuditschko, Herman W. Raadsma, Mehar S. Khatkar, Elisabeth Jonas, Eike J. Steinig, Christine Flury, Heidi Signer-Hasler, Mirjam Frischknecht, Ruedi von Niederhäusern, Tosso Leeb, Stefan Rieder

**Affiliations:** 1 Agroscope, Swiss National Stud Farm, Avenches, Switzerland; 2 Reprogen – Animal Bioscience Group, Faculty of Veterinary Science, University of Sydney, Camden, Australia; 3 SLU, Department of Animal Breeding and Genetics, Uppsala, Sweden; 4 College of Marine and Environmental Sciences, James Cook University, Townsville, Australia; 5 School of Agricultural Forest and Food Sciences, Bern University of Applied Sciences, Zollikofen, Switzerland; 6 Institute of Genetics, Vetsuisse Faculty, University of Bern, Bern, Switzerland; Estonian Biocentre, ESTONIA

## Abstract

Evaluating the genetic contribution of individuals to population structure is essential to select informative individuals for genome sequencing, genotype imputation and to ascertain complex population structures. Existing methods for the selection of informative individuals for genomic imputation solely focus on the identification of key ancestors, which can lead to a loss of phasing accuracy of the reference population. Currently many methods are independently applied to investigate complex population structures. Based on the Eigenvalue Decomposition (EVD) of a genomic relationship matrix we describe a novel approach to evaluate the genetic contribution of individuals to population structure. We combined the identification of key contributors with model-based clustering and population network visualization into an integrated three-step approach, which allows identification of high-resolution population structures and substructures around such key contributors. The approach was applied and validated in four disparate datasets including a simulated population (5,100 individuals and 10,000 SNPs), a highly structured experimental sheep population (1,421 individuals and 44,693 SNPs) and two large complex pedigree populations namely horse (1,077 individuals and 38,124 SNPs) and cattle (2,457 individuals and 45,765 SNPs). In the simulated and experimental sheep dataset, our method, which is unsupervised, successfully identified all known key contributors. Applying our three-step approach to the horse and cattle populations, we observed high-resolution population substructures including the absence of obvious important key contributors. Furthermore, we show that compared to commonly applied strategies to select informative individuals for genotype imputation including the computation of marginal gene contributions (Pedig) and the optimization of genetic relatedness (Rel), the selection of key contributors provided the highest phasing accuracies within the selected reference populations. The presented approach opens new perspectives in the characterization and informed management of populations in general, and in areas such as conservation genetics and selective animal breeding in particular, where assessing the genetic contribution of influential and admixed individuals is crucial for research and management applications. As such, this method provides a valuable complement to common applied tools to visualize complex population structures and to select individuals for re-sequencing.

## Introduction

Recent innovations in high throughput sequencing [1] and array technologies [2] have led to the development of draft/reference genomes for an extensive range of domestic animal species and the identification of large numbers of single nucleotide polymorphisms (SNPs) [3–5]. Presently, global efforts are focusing on re-sequencing additional animals within species and breed groups to improve knowledge on the genetic architecture and allow identification of high-resolution variation between individuals [6–9]. A typical approach in such scenarios is to re-sequence informative individuals within populations, and to impute whole genome sequence level genotypes of additional animals genotyped with high density SNP panels [10, 11].

Existing methods for the selection of reference individuals for genotype imputation solely focus on the identification of key ancestors through pedigree or genomic relationship information to maximize genetic diversity [12, 13]. Typically such strategies do not account for population substructures and neglect the genotype information of the most influential progeny, which leads to a loss of phasing accuracy of the reference population [14, 15] and has posed problems in genotype imputation [15]. Therefore, we propose an alternative strategy to select informative individuals within genotyped populations based on the Eigenvalue Decomposition (EVD) of a genomic relationship matrix among genotyped individuals.

Eigenvalue Decomposition like Principal Component Analysis (PCA) is a multivariate technique that provides an optimal subspace to investigate population structures by maximizing variation on the highest ranked components [16]. Based upon this mathematical principle we identified individuals that maximizes the variation of the genetic relationship structure accounted for, by calculating the correlation between each individual and the number of significant components. Individuals that capture most of the variation in the relevant genetic relationship structure within populations will hereafter be referred to as “key contributors”. Here, we demonstrate that the identification of key contributors is directly associated with the number of significant components and that the selection of key contributors increases phasing accuracy of the reference populations compared to other commonly applied methods. Of note is that key contributors are distinctly different from the identification of key ancestors through pedigree or genomic relationship information to maximize genetic diversity [12, 13] as we show further on in the results.

Recently, it was demonstrated that population substructures due to admixture affect imputation reliability and accuracy [11, 17]. Commonly, model-based algorithms such as implemented in Structure [18] and Admixture [19], as well as distance-based methods derived from PCA [20] are applied to uncover population substructures. The results of numerous studies show that both methods are efficient tools to investigate population structures based on genome-wide SNP data [21–23]. However as such, these methods do not provide any information on the genetic contribution of individuals within populations. Therefore, we combined the identification of key contributors with model-based clustering (Admixture) [19] and high-definition network visualization (NetView) [24] into an integrated three-step approach, which supports the investigation of complex population structures without the knowledge of *a priori* ancestry information. Furthermore, we discuss how the results of this study can be used to maintain genetic diversity in breeding and conservation programs, to allow identification of substructures for downstream analyses such as genome-wide association (GWA), genomic selection (GS), and genome re-sequencing studies.

## Materials and methods

### Computation of genetic relationship matrices

The identification of key contributors is based on the EVD of a relationship matrix and the number of significant components. This requires as input a symmetric relationship matrix **A** of dimension *n* x *n*, where *n* is the number of individuals. To account for Mendelian sampling effects within the populations we decided to use identity by descent (IBD) genomic relationship matrices (**G**) computed using Germline [25]. Germline was run with default parameter setting except for “-bits 9” and “-err hom 1”, using the phased haplotype data on all the autosomes as input data. The haplotype data of the three livestock populations were inferred using Dualphase [26] in case of sheep, whilst haplotypes for horse and cattle were derived with the software package Beagle v3.3.2 [27]. For the simulated data we used known haplotypes. The IBD segments were computed among all pairs of individuals, and the pair-wise sum of these segments expressed as a proportion of the total length of the autosomal genome, was taken as the IBD genomic relationship. However, it is important to note that different kinds of genetic relationship matrices [28, 29]can also be applied.

### Determining the number of significant components

To determine the number of *k* significant components for **G** we used the empirical method described as Horn’s parallel analysis, which is implemented in the statistical software package *paran* (http://www.r-project.org). This method employs Monte Carlo estimates to retain the most significant components under a defined level of significance and number of iterations. Here, we chose a significance level of P = 0.01 and 10,000 iterations, which have been suggested in the modified version of Horn’s parallel analysis [30].

### Identification of key contributors

The EVD of **G** returns *n* nonnegative eigenvalues *λ*_*i*_, *i* = 1, …, *n* and *n* singular eigenvectors **u**_*i*_, *i* = 1, …, *n*. Traditionally, the set of **u**_*i*_ are summarized in the matrix **U**, where each column corresponds to an eigenvector and *λ*_*i*_ are stored in a diagonal matrix **λ** such that:

$$G\;=U\;\lambda \;U^{T}\;\lambda =diag\;(\lambda {\;}_{1}\;,\ldots ,\;\lambda {\;}_{n}).$$ (1)

Inferring eigenvectors or principal components (PCs) is a common strategy in population genetics to visualize population structures based on a small number of PCs (e.g. two or three) and to allocate individuals to population clusters on low dimensional data [28]. As such, eigenvectors are mathematical abstractions and do not correspond to an individual. To determine which individuals lie in the optimal subspace spanned by the top *k* significant components, we derived standardized eigenvectors by dividing the eigenvectors by the square root of their corresponding eigenvalues $\left(s_{i}=\frac{u_{i}}{\sqrt{\lambda_{i}}}\right)$, which is a common procedure in Principal Coordinate axes analysis (PCoA) [31]. Next, we calculated the correlation (**r**_*ij*_) between the *j*—th individual (**g**_*j*_) and the *i*—th standardized eigenvector (**s**_*i*_) limiting the number of **s**_*i*_ to the first *k* significant components (see description above)

$$r_{ij}\;=\;s_{i}^{T}g_{j}.$$ (2)

Finally, we ranked all individuals according to the genetic contribution score (*gc*_*j*_) and considered individuals correlated with top *k* significant components as key contributors

$$gc_{j}\;=\;\sum_{i=1}^{k}{(r_{ij})}^{2}.$$ (3)

The method to determine key contributors within populations is implemented in R (http://www.r-project.org) and available online at https://github.com/esteinig/netview.

### Admixture

To estimate the individual levels of admixture (*a*_*j*_) within the two admixed populations (sheep and horse) we performed model-based cluster analyses using the program Admixture 1.23 [19]. We ran Admixture assuming two ancestral populations (K = 2) in 100 replicates and assessed convergence between individual runs. Using low values for K, all runs arrived at the same or very similar log-likelihood scores (LLs). Admixture results were integrated within the high-resolution population structure analyses and also visualized with the program Distruct 1.1 [32].

### Netview

**Fig 1:**
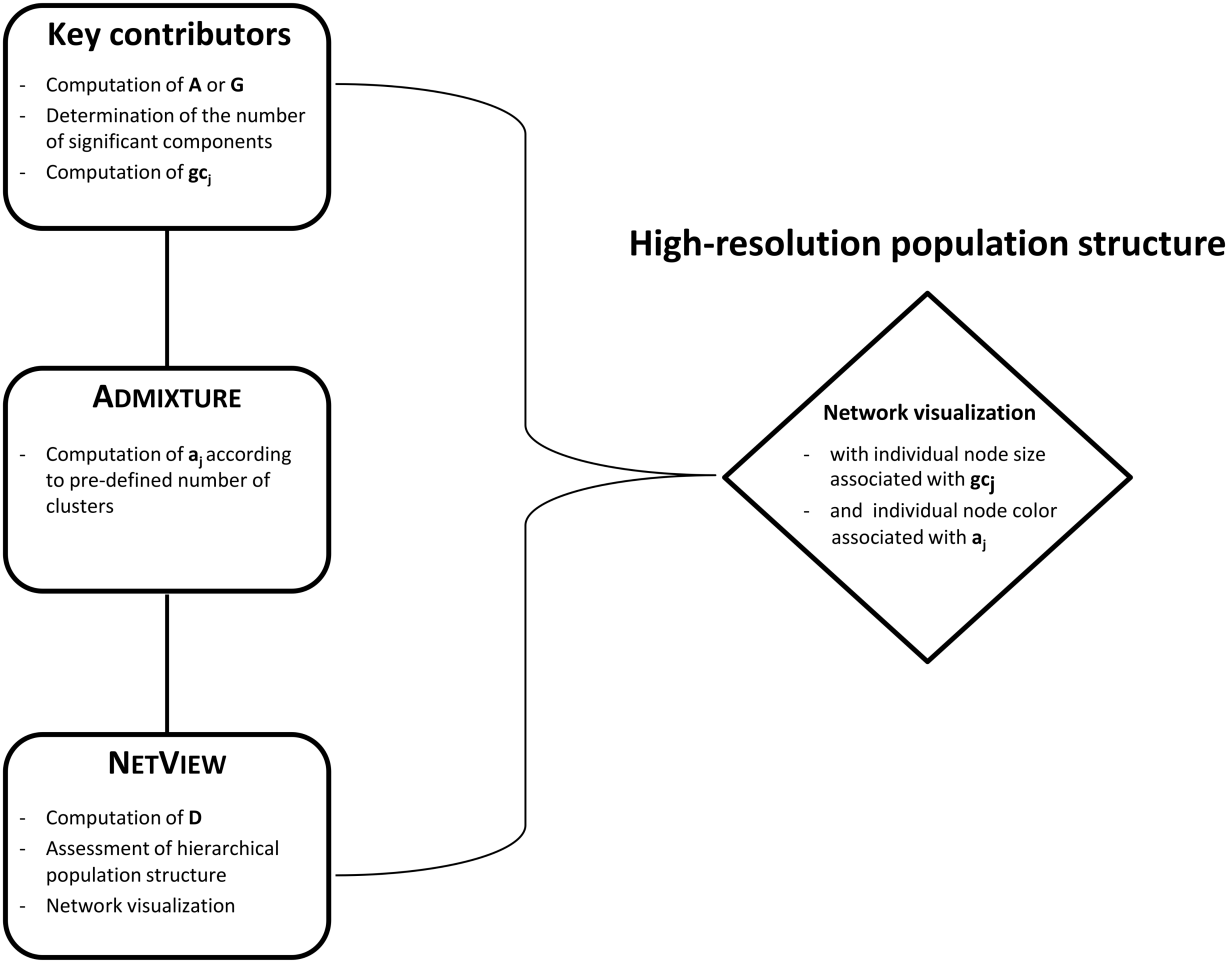
Workflow of the high-resolution population structure analysis. Schematically representation of the different analyses involved in the integrated three-step procedure.

To identify and visualize *a*_*j*_ and *gc*_*j*_ of the individuals within the populations, we used the network-based clustering algorithm Spc [33] as implemented in the high-definition network visualization approach NetView [24]. The input to Spc is a symmetric distance matrix (**D**) between individuals, with genetic distances for all samples being calculated by subtracting pairwise relations from one (1 − **G**). The free parameters of Spc are the numbers of *k*-nearest neighbors (*k*-NN), a Pott spin variable (*q*) and the range of temperature along which the clustering is performed (*ΔT*). We applied the algorithm in its default setting, that is *k*-NN = 10, *q* = 20 and *ΔT* = 0.01. An implementation of NetView pipeline is also posted at http://sydney.edu.au/vetscience/reprogen/netview/ and was recently described as a Python pipeline by Steining *et al*. [34] https://github.com/esteinig/netview. In order to retain well-structured population networks, we used the open graph visualization platform Cytoscape v.2.83 [35] and the plugin *MultiColoredNodes* [36] for the final network visualization. Applying NetView population structure is presented in terms of nodes, edges between nodes and thickness of edges. In the final network presentation, we have associated the node size of each individual with their respective *gc*_*j*_, whilst the node color of each individual represents the proportion *a*_*j*_ according to the pre-specified number of clusters (see workflow as illustrated in Fig 1). In order to express the strength of relationship between individuals the thickness of an edge is associated with the genetic distance between two nodes, with thicker edges corresponding to lower genetic distance.

### Phasing accuracy of selected reference populations

To demonstrate the utility of our strategy for selecting key contributors in complex population structures to increase phasing accuracy of the respective reference population, we compared the phasing accuracy of selected individuals with two other methods suggested in the literature [14, 37]. For comparison we additionally identified sets of individuals selected based on their pedigree-based marginal gene contributions using the program package Pedig (Ped) [12], expected genetic relationships to the reference population as presented by Goddard and Hayes (Rel) [13] and animals selected at random (Ran). Here we also applied Rel strategy on **G** as described above. After selecting sets of informative individuals according to the respective methods, the inferred haplotype phase of the simulated data was compared with the true haplotype phase for each individual in the reference population, whilst for sheep, horse and cattle we used the most likely haplotype phase. For this purpose we phased individual genotypes of the three populations based on the given pedigree structure including the information of trios and duos. These haplotypes should be highly accurate and hence suitable for validation. We examined phasing accuracy by using the switch-error-rate metric, dividing the number of observed switches by the number of all heterozygous SNPs-1 [38]. Phasing accuracy was evaluated for the different sets of key contributors in each population and four additional scenarios increasing the number of selected individuals from 20 to 80 in increments of 20. Phasing of the three livestock populations and the selected sets of reference individuals was performed with the program FImpute [39].

### Simulated data

The simulated data consisted of a total of 5,100 individuals and 10,000 SNPs as described by Usai *et al*. [40] at http://qtl-mas-2012.kassiopeagroup.com/en/dataset.php. The simulation starts with a base population (F0) of 1,020 unrelated individuals (20 males and 1,000 females). The first generation (F1) was generated by randomly mating each of the 20 founder males with 50 females. All dams produced female offspring, except 20 dams which generated two offspring (one male and one female offspring). Each of the next three generations (F2-F4) also consisted of 20 males and 1,000 females and was generated following the same principle, by randomly mating each male with 50 females of the previous generation, whilst the five generations did not overlap. The simulated genome consisted of five chromosomes each spanning 100 Mb with 2,000 equally distributed SNPs. Applying a random mating strategy in the simulation it is possible that inbreeding occurs within each of the following generations (F2-F4). Therefore, we computed the genome-based inbreeding coefficient of all individuals (*f*) using the software package Plink [41].

### Sheep data

The sheep data represents an experimental backcross/intercross sheep resource flock. The mating strategies and development of this population were described in detail by Raadsma *et al*. [42]. Briefly, the establishment of the sheep population was done in three phases. In phase one, F1 males and females were produced by crossing four Awassi founder sires (F0) with 30 fine/medium wool Merino ewes. Four F1 sires were selected to represent each of the F0 sires as well as one medium wool Merino (F1 Sire_1) and three related fine wool Merinos F0 dams (F1 Sire_2, F1 Sire_3 and F1 Sire_4). After selection, these four F1 sires were backcrossed to unrelated medium wool Merino ewes. In total each F1 sire had 488, 313, 279, and 126 progeny. In phase two, backcross ewes were mated to F1 sires and in phase three backcross ewes and backcross sires were intercrossed to produce F2 (intercross) progeny. In phase two additional three F2 sires were selected for mating, reproducing a total of 89, 67 and 48 progeny. Here, we studied 1,421 individuals from this resource population including backcross (BC), double backcross (DBC) and intercross (INT) progeny as well as the seven selected sires (four F1 sires and three F2 sires). The animals were genotyped using the Illumina OvineSNP50 BeadChip^®^ covering 54,241 genome-wide SNP genotypes. Quality Control (QC) filters were applied, removing SNPs with call rate less than 90%, those with very low minor allelic frequency (MAF) <0.05 and SNPs showing a number of mismatches between paternal and offspring genotypes. Post QC we retained 44,693 SNPs located on all autosomes and genotypes on 1,421 sheep. To calculate the level of admixture of sheep we included the genotype information of the four Awassi founder sires (F0) and 25 Merinos in the reference population (six founder Merinos (F0) and 19 most unrelated Australian Industry Merinos). The SNP genotypes of the 19 most unrelated Australian Industry Merinos were derived from the International Sheep Genomics Consortium (ISGC) [22] (http://www.sheephapmap.org).

### Horse data

The horse data consisted of sample collection of 1,077 horses previously described by Signer-Hasler *et al*. [43]. This dataset was selected to represent an active breeding population including stallions with many offspring, younger stallions and breeding mares of the Swiss Franches-Montagnes (FM) horse breed. The population structure of this breed is based upon the formation of 11 major stallion lineages, where especially three of these lineages show a high level of admixture with Arabian and Warmblood horses, respectively. In order to determine the level of admixture of crossbred FM horses, we additionally included the SNP genotype information of 600 Warmblood horses [44] in the data. Post QC (MAF >0.05, call rate >0.9 and Hardy Weinberg Equilibrium (HWE) P >0.0001) we included 38,124 SNP genotypes of the FM horses for the final analyses. In addition, we derived the genotype information of un-genotyped key contributors using the software package FImpute [39].

### Cattle data

Finally, we applied our method on 2,457 progeny-tested Australian Holstein-Friesian bulls [45] genotyped with the Illumina Bovine SNP50 BeadChip [46]. The majority of these bulls (2,420) were born between 1980 and 2007, whilst 37 bulls were born before 1980. After applying QC filters (MAF >0.01, call rate >0.9 and Hardy Weinberg Equilibrium (HWE) P >0.0001) a total of 45,765 autosomal SNPs were included in the analyses.

## Results

### Simulated data

**Fig 2:**
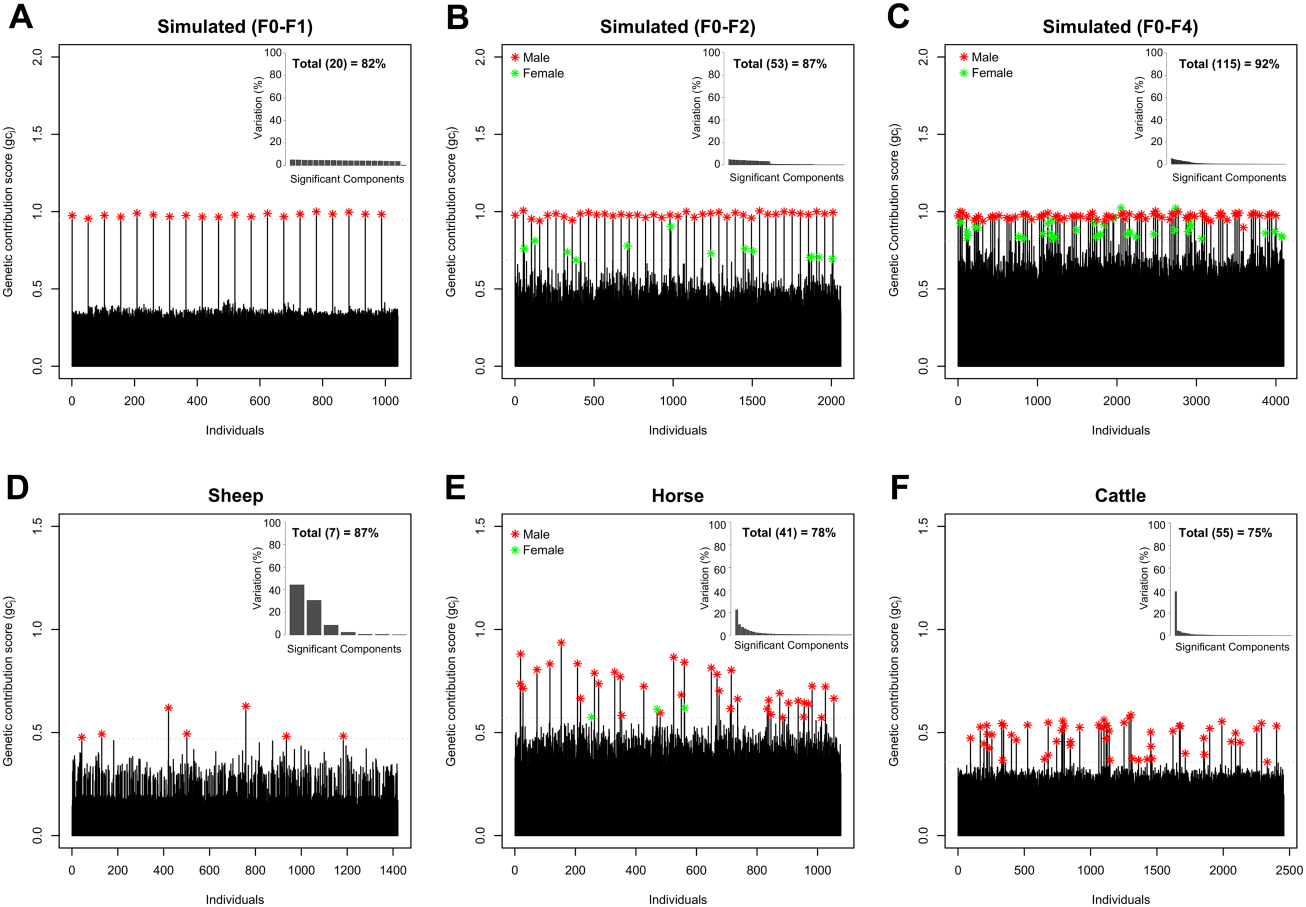
Identification of key contributors within the four populations. Proportion of variation corresponding to the number of significant components and genetic contribution scores (*gc*_*j*_) of each selected dataset (A-F). Top key contributors according to the number of significant components are indicated by red (male) and green stars (female), respectively.

Analysis of the 20 founder males (F0) and the respective progeny of the F1 generation (1,020 individuals), resulted in 20 significant components, which accounted for 82% of the variation of the genetic relationship structure (Fig 2A, top right). Based on the optimal number of significant components, *gc*_*j*_ were computed for each individual (using Eq 3). The distribution of *gc*_*j*_ illustrates that the 20 founder males were clearly identified within the F1 generation and suggests that the remaining individuals did not make a significant genetic contribution to account for the genetic variation of the population relationship structure (Fig 2A, red stars). Including the individuals of the F2 generation in the analysis, 53 significant components accounting for 87% of the variation of the genetic relationship structure were determined (Fig 2B, top right) and the 40 contributing males of the first two generations (F0-F1) were identified as top key contributors based upon *gc*_*j*_ (Fig 2B, red stars). Extending the same analysis to all generations (F0-F4), resulted in 115 significant components, which accounted for 92% of the variation of the genetic relationship structure (Fig 2C, top right). Ranking the individuals according to *gc*_*j*_ showed that all the 80 contributing males (F0-F3), and 35 females (F1-F4) were included in the selection of top 115 individuals (Fig 2C, red and green stars). Comparing the inbreeding coefficient (*f*) of the 35 females to the remaining population reveals that these females are highly inbred (S1 Fig).

### Sheep data

For the sheep dataset, seven significant components accounted for 87% of the variation of the genetic relationship structure. (Fig 2D, top right). The distribution of *gc*_*j*_ showed that compared to the simulated population structure, only a few sheep contribute to the variation of the genetic relationship structure (Fig 2D, red stars). Ranking the top seven key contributors according to *gc*_*j*_ clearly identified the seven foundation sires (four F1 sires and three F2 sires) and simultaneously revealed that two F2 sires were the most influential individuals within the population (S1 Table). Both F2 sires are highly inbred rams (DBC) with foundation sire F1 Sire_1 and F1 Sire_2 being its sire and grand-sire, respectively. The third F2 sire, which descended from F1 Sire_4 (sire) and F1 Sire_3 (grand-sire) was ranked on 6^th^ position. Within the four F1 sires the ranking indicates that F1 Sire_1 descending from a different medium wool Merino strain compared to the other three F1 sires (derived from superfine wool Merino dams), and F1 Sire_3, which was not used in the production of the intercross progeny (INT), were less influential.

**Fig 3:**
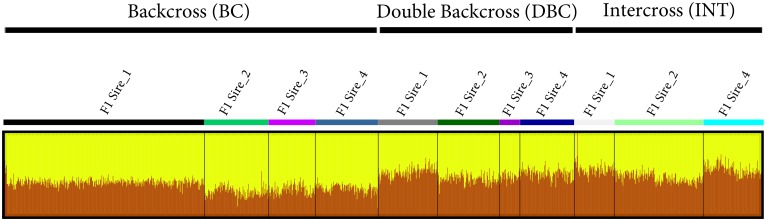
Admixture of experimental sheep. Cluster assignment assessed with Admixture at K = 2. Individuals are presented by single vertical column, whilst the length of the colored segment represents the estimated level of admixture (Awassi = brown; Merino = yellow).

**Fig 4:**
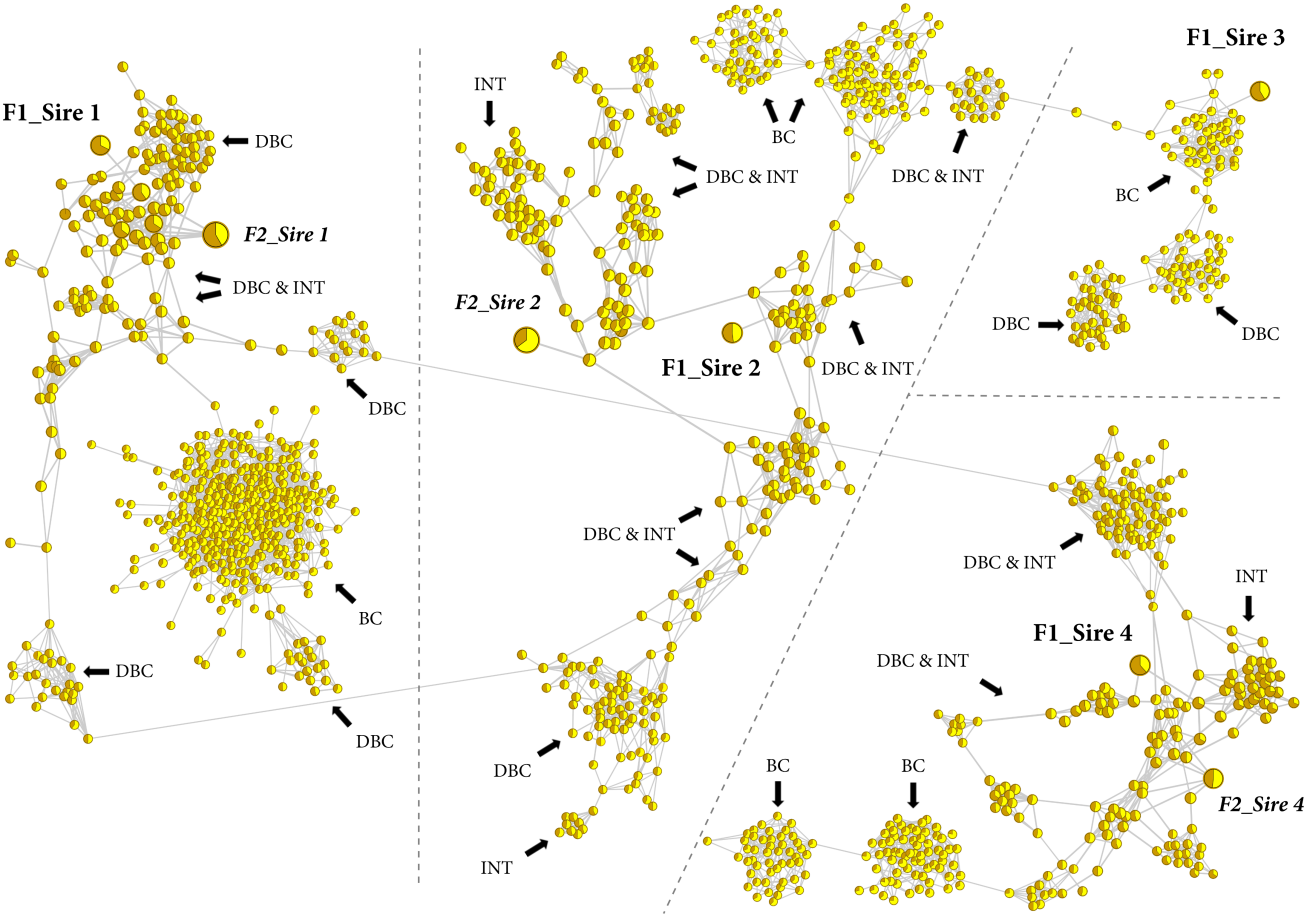
High-resolution population structure of experimental sheep. Network visualization of 1,421 sheep. Each sheep is represented by a node; with individual node size associated with *gc*_*j*_, whilst the different node colors represent *a*_*j*_ between Awassi (brown) and Merino (yellow). Top seven key contributors are represented by an increased node size. The thickness of edges varies in proportion to the genetic distance to visualize individual relationships within the population. The progeny of the four different F1 sires are separated by dashed lines, whilst the different progeny cluster: backross (BC), double backcross (DBC) and intercoss (INT) are denoted by an arrow.

To further examine the structure of the sheep population, we computed *a*_*j*_ for each sheep and performed a high-resolution population structure analysis. Dividing the sheep into groups of the applied mating strategies (BC, DBC, INT) and subgroups of the four different F1 sires showed that DBC and INT animals share the same admixture pattern, whilst BC animals show a distinct level of admixture (Fig 3). Comparing *a*_*j*_ between the four F1 progeny subgroups additionally illustrates that especially BC and DBC animals of F1_Sire 1 have an increased level of admixture with Awassi compared to the progeny of the other three F1 sires. Finally we integrated *gc*_*j*_ and *a*_*j*_ in the population network visualization, with the node size of each individual associated with *gc*_*j*_, and node color associated with *a*_*j*_. The high-resolution population network structure clearly separates the sheep into well-defined population clusters according to the applied mating strategies (BC, DBC and INT) and simultaneously highlights the existence, of the seven sires selected for the mating design (Fig 4). Besides the detection of the seven foundation sires, the network visualization further represents the level of admixture in the respective population clusters and thereby reveals that especially highly admixed Awassi animals (INT and DBC) were associated with high *gc*_*j*_, whilst highly admixed predominant 75% Merino (BC) animals were generally associated with lower *gc*_*j*_.

### Horse data

**Fig 5:**
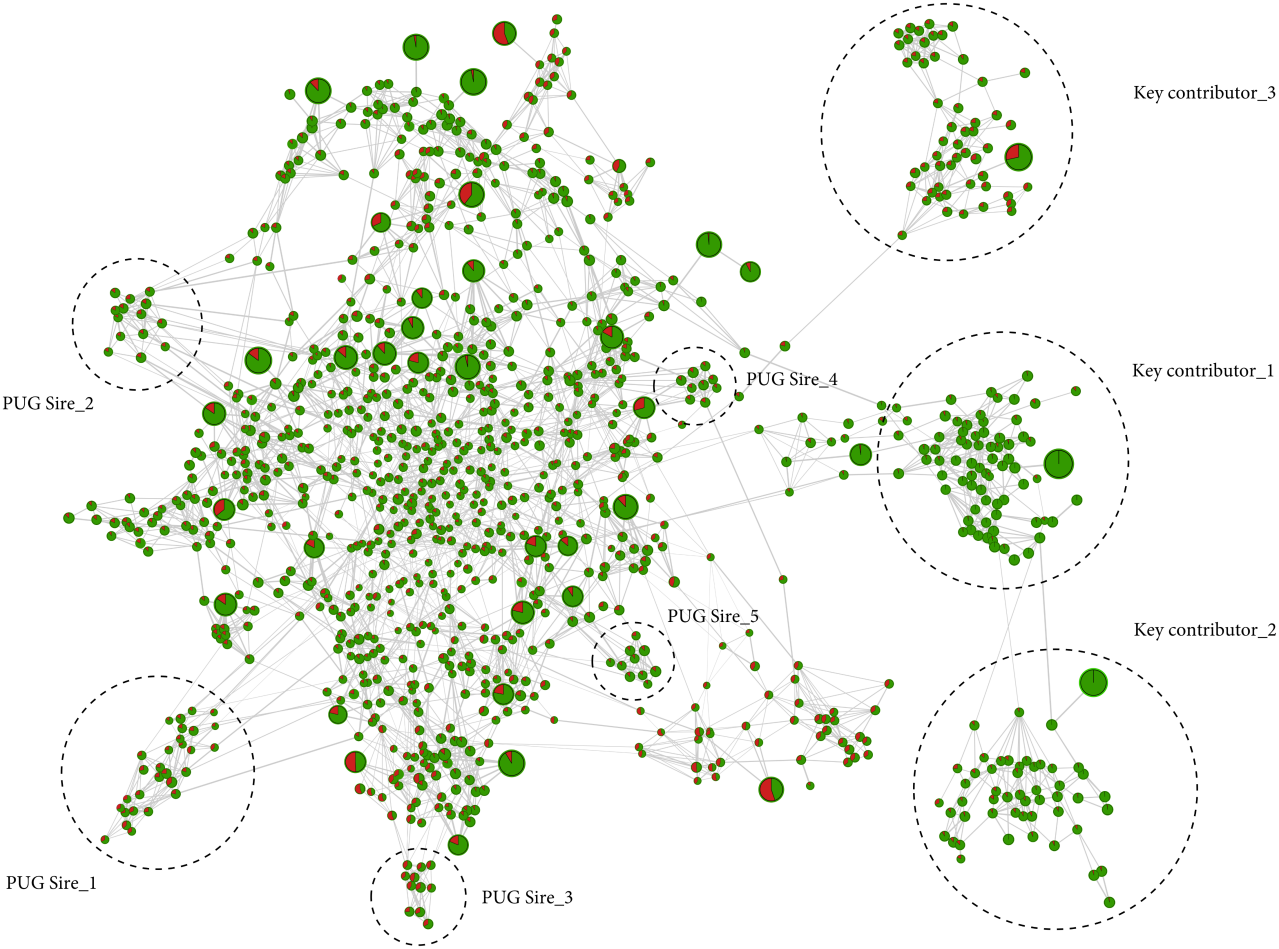
High-resolution population structure of horse. Network visualization of 1,077 horses. Each horse is represented by a node; with individual node size associated with *gc*_*j*_, whilst the two different node colors represent *a*_*j*_ between Swiss Franches-Montagnes (FM) (green) and Warmblood (red). Top 41 key contributors are represented by an increased node size. The thickness of edges varies in proportion to the genetic distance to visualize individual relationships within the population. The topology of the network reflects the population structure of the FM horse breed and reveals sub-structures caused by the progeny of most influential stallions. The progeny clusters of the three most influential stallions and un-genotyped sires (PUG) are indicated by a dashed circles.

For the horse dataset, 41 significant components accounted for 78% of the variation of the genetic relationship structure (Fig 2E, top right) and especially stallions frequently used for reproduction were assigned with high *gc*_*j*_ (Fig 2E, red stars). The high-resolution population network splits the horses into four distinct population clusters, whilst the progeny of the three most influential stallions were assigned into three distinct population clusters (Fig 5, dashed circles). The split into four distinct clusters appears to correspond to the known population structure of the FM horse population, where in particular these three stallions were used extensively in recent breeding history. The most evident substructures at the edge of the network of the remaining horses corresponded to the most influential sires and their progeny, whilst less influential individuals were assembled in the center of the network. The topology of the network additionally illustrates the impact of crossbred FM horses on the formation of the population and revealed that within some population clusters no key contributors could be identified (Fig 5, dashed circles). Further investigation of ancestry information of these population clusters, showed that their common ancestors were not genotyped (PUG Sire). In order to evaluate the contribution of these ancestors to the population structure, we imputed the genotypes of un-genotyped sires based on the genotype information of their progeny including nine up to 29 progeny per sire. After determining the correlation between the non-genotyped sires and the 41 significant components, all five ancestors were ranked amongst top key contributors (S2 Fig).

### Cattle data

**Fig 6:**
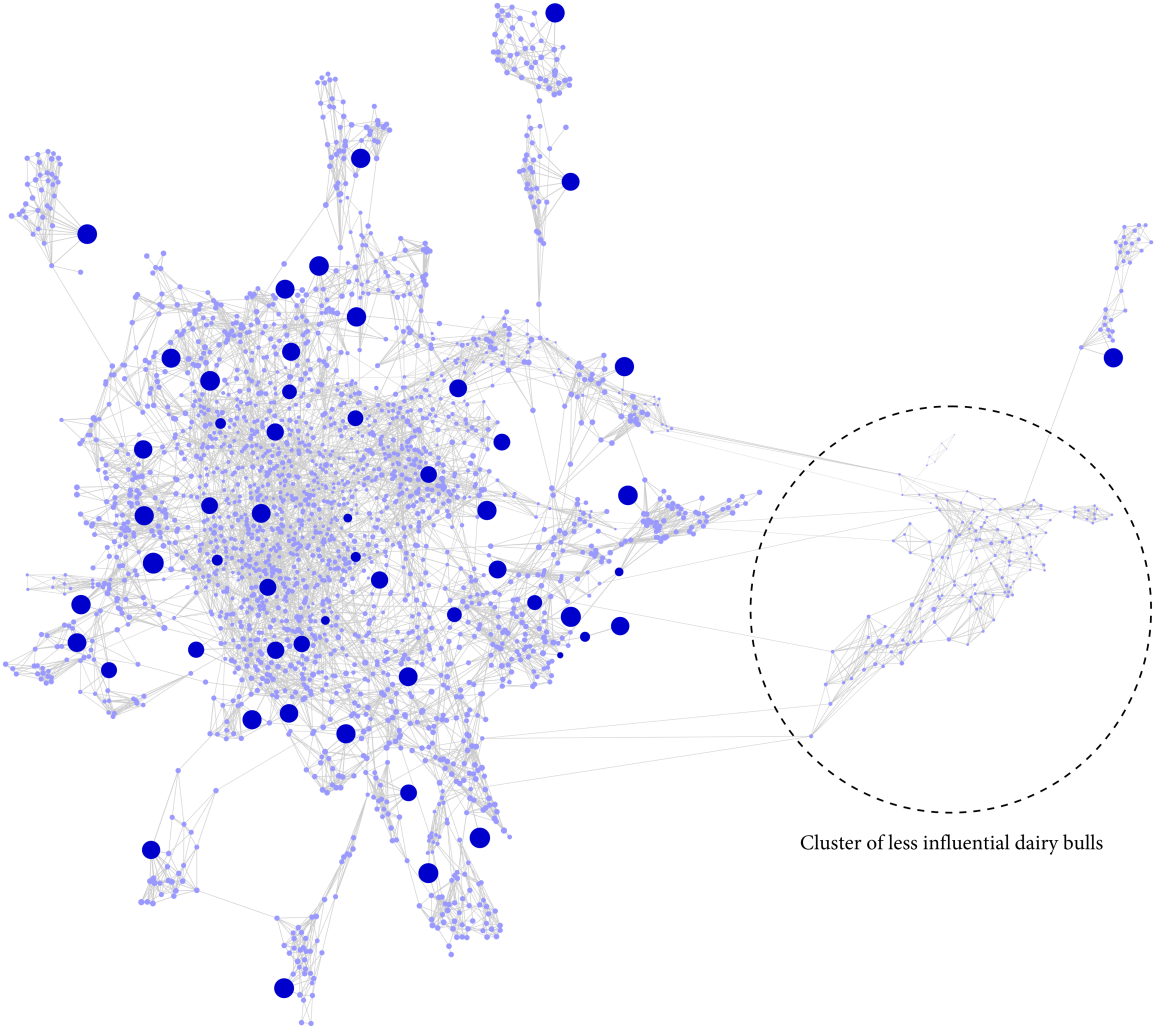
High-resolution population structure of cattle. Network visualization of 2,457 cattle. Each cattle is represented by a node; with individual node size associated with *gc*_*j*_, whilst the node color (dark blue) indicates top 55 key contributors. The thickness of edges varies in proportion to the genetic distance to visualize individual relationships within the population. The network structure of indicates that key contributors are well distributed within the population and highlights the existence of a substructure according to less influential bulls (dashed circle).

For cattle, 55 significant components accounted for 75% of the variation of the genetic relationship structure (Fig 2F, top right) and it could be shown that 55 key contributors stood out from all other individuals (Fig 2F, red stars). The high-resolution network illustrates that, like horse, key contributors and their respective progeny were assigned into distinct population clusters and accounted for much of the population stratification (Fig 6). Based on *gc*_*j*_ it can be further noted that the top key contributors are well-distributed over the whole population and that a cluster of less influential dairy bulls caused an additional substructure within the data (Fig 6, dashed circle). This result is also supported by a reordered heat map of **D** according to the network based population structure (S3 Fig). The ancestry information of these less influential bulls showed that they were born between 1955 and 1998 and as such did not make a significant contribution to the more recent sample collection (1990–2007).

### Phasing accuracy of selected reference populations

**Fig 7:**
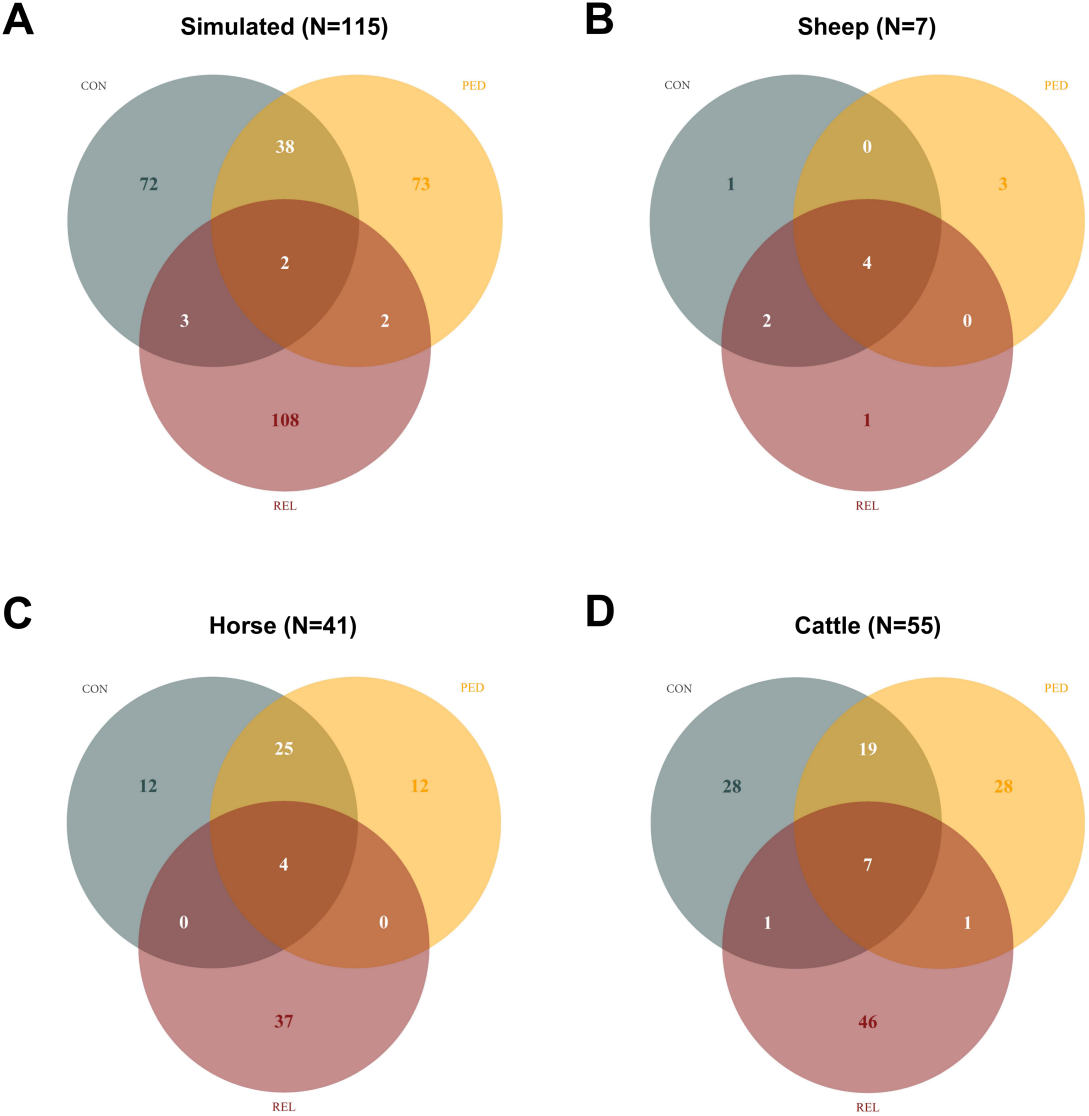
Overlap between informative individuals using three different selection strategies. Venn Diagrams representing the overlap between the three different strategies (Con, Rel and Ped), when selecting top key contributors in each population.

Comparing top key contributors (Con) of each population with sets of individuals selected under Rel and Ped showed that, with the only exception of sheep, Ped and Con shared the most common individuals in the selected reference populations (Fig 7A–7D). The overlap between the three strategies additionally reveals that Rel failed to identify the 20 founder males within the simulated dataset, whilst within sheep all three applied methods successfully allocated the four F1 foundation sires. Furthermore we have noticed that, within cattle the selected subsets under Rel and Ped included 10 and 9 of the less influential bulls, respectively (see Fig 6, dashed circle). Table 1 shows the switch error rate of the selected reference populations in each dataset, including respective sets of individuals selected at random (Ran). Selected reference populations under Con consistently resulted in the most accurate haplotypes of the applied selection strategies within all datasets. The simulated reference populations had a mean switch error rate of 0.35% (Con) followed by 0.41% (Ped) and 4.27% (Rel), whilst Rel performed worse than selecting random (Ran) individuals (1.39%). The sheep dataset stands out here, as all selected reference populations showed very accurate haplotypes (switch error rate < 0.40%), which can be explained by the highly structured mating design. Within the horse dataset, again reference populations selected under Con had the lowest mean switch error rate (0.62%) followed by Ped (0.74%) and Ran (0.97%). Once again Rel (1.52%) performed worse than the other three methods. Compared to the other three populations the selected reference populations of cattle showed the highest switch error rates ranging between 1.64% (Con) and 3.48% (Rel).

**Table 1:** Switch error rates of the selected reference populations within the four populations.

Strategy	Simulated (N = 115)	Sheep (N = 7)	Horse (N = 41)	Cattle (N = 55)
Con	0.35%	0.26%	0.62%	1.64%
Rel	4.27%	0.31%	1.52%	3.48%
Ped	0.41%	0.27%	0.74%	2.10%
Ran	1.39%	0.94%	0.97%	1.70%

Selecting additional subsets including 20 to 80 individuals in the reference population shows that the accuracy of haplotypes increased as the number of informative individuals increased within all reference populations and highlights that phasing accuracy of the selected reference populations is directly related to the respective population structure, as especially smaller subsets of the simulated population had the highest switch error rates compared to horse and cattle (S4 Fig). Furthermore, the results illustrates that even though smaller subsets than identified key contributors were selected, the reference populations determined by Con still resulted in the most accurate haplotypes.

## Discussion

The purpose of this study was to identify key contributors in complex population structures to increase the resolution of population structure analyses and to evaluate the utility of using such key contributors to increase phasing accuracy of selected reference populations. To date, the two popular methods of choice for the identification of population structure, sub-division and admixture are parametric (e.g. Admixture) [19] and non-parametric techniques (e.g. PCA) [20]. Recently, network-based cluster approaches are regaining favor for uncovering population structures like NetView [24, 34]. We extended the recent NetView approach with Admixture [19] and the identification of the key contributors into an integrated three-step procedure that provides a high-resolution analysis and visualization of population structures (Fig 1).

We exemplified the three-step procedure in four diverse datasets to highlight its unique features. The first two datasets consisted of a simulated population structure and an experimental backcross/- intercross resource flock. The highly structured design of these two datasets allowed us to validate the method and to determine the number of contributing individuals in such populations. The other two datasets, namely the horse and cattle population, represent a complex population structure with major founder lineages. Such datasets are common in livestock and other breeding schemes where systematic breeding decisions are made for mate allocation over a long period of time. Once again our procedure successfully determined the most influential animals, indicated the absence of obvious important key contributors (Fig 5) and detected population stratification of less related individuals (Fig 6). Furthermore, we identified fine-scale differences in ancestry profiles between individuals by including admixture analyses within the sheep and horse dataset. Therefore, our procedure can be thought of as a complement to the aforementioned methods for the identification of population structures [19, 20]. These latter methods (PCA and Admixture) are powerful to separate individuals into relatively homogeneous population clusters, and simultaneously revealing sub-structures and genetic outliers within the populations. However, these methods do not provide any information on the genetic contribution of the individuals and are less-suited to visualize high-resolution population structures of very large datasets [24]. The three-step procedure presented here is designed to more directly address these important questions.

Compared to commonly applied methods used to select informative individuals for genotype imputation [47], key contributors are most likely associated with major population clusters (Fig 6), regardless of their genetic relatedness. Thus, with the application of key contributors it becomes feasible to include influential progeny in the selection of a reference population and to determine if individuals originating from specific lineages/strains had a great impact on the formation of the population. The phasing results (Table 1) clearly demonstrate that including key contributors in the selection of an optimal reference population increases phasing accuracy, which is particularly beneficial for small reference populations [14]. With increasing numbers of individuals in the reference population, we strongly recommend to remove redundancy of the selected key contributors by including the genetic relatedness of the individuals in the analysis. However, it should be noticed that the best phasing accuracy is always given by including all available individuals in the reference population. We observed large differences in phasing accuracy between the tested populations when applying the different methods. Several factors may explain these differences, such as data composition, genetic diversity and substructures [11]. We suspect that the low phasing accuracies in cattle are caused by the substructure of less related bulls (Fig 6) and that the genotype information of contributing dams were not included in the dataset, leaving the cattle reference population underrepresented compared to the other three populations. In order to improve phasing accuracy of the reference population we suggest to also include contributing dams in the data collection and to scan for population substructures and missing key contributors prior the selection of informative individuals for genotype imputation. Therefore, the four steps involved in an optimal selection of informative individuals for phasing and genotype imputation are:

1. Select a set of samples that maximizes the variation attributed to the most significant components by including most important key contributors.
2. Identify key contributors according to the number of significant components.
3. For large reference populations, perform a high-resolution network visualization to remove putative redundancy of selected key contributors.
4. Perform phasing of the reference population involving key ancestors and influential progeny (key contributors) prior imputation.

Besides phasing accuracy, the identification of key contributors is also relevant to other research scenarios, such as genetic diversity and conservation genetics. For instance, the identification of key contributors can be especially useful to study indigenous and endangered populations hereby providing essential information on the formation and the management of small populations. In order to conserve genetic diversity the identification of high-resolution population structures based on key contributors can be used as an optimal monitoring tool to avoid inbreeding and to evaluate the genetic development of populations over a period of time, despite missing ancestry information in many, especially wild-life or indigenous populations (see the high-resolution population structure of a pearl oyster population at https://github.com/esteinig/netview).

The integrated three-step procedure can be easily applied to investigate population structures of very large datasets including many thousands of individuals. Perhaps the most important advantage of the method is that none of the three steps involved rely on *a priori* assumptions or modeling of the data. Key contributors are simply detected by determining the correlation of the individuals based upon the number of significant components, whilst model-based clustering and NetView can be applied to examine the level of admixture and genetic relatedness of key contributors, hereby providing a high-resolution population structure of the data. However, it is important to note that NetView can be combined with any other model-based clustering approach (e.g. Structure [18]) and any other strategy to identify informative individuals within populations [12, 13].

We have described modifications based on existing principles and methods commonly used in the analysis of complex data structures to investigate population structures using genome-wide SNP information. Firstly, we describe the identification of key contributors within complex population structures. Secondly, we were able to select key contributors that increased phasing accuracy within small reference populations. Finally, with the combination of the identification of key contributors, model-based clustering and NetView we present a novel three-step approach that can provide new insights into high-resolution population structures at low levels of genetic differentiation. Therefore, we believe that the identification and visualization of key contributors within populations will be of invaluable benefit for geneticists to investigate complex population structures.

## Supporting information

S1 File Simulated data. Pedigree, genotype and IBD information of the simulated data.
(ZIP)

S1 Fig Inbreeding coefficient (*f*) of selected females within simulated data. Boxplots, which indicate the median value, 25% and 75% quartiles of the inbreeding coefficient of the 35 selected females (blue) and the remaining population (grey).
(PDF)

S2 Fig High-resolution population structure of horse. Network visualization of 1,082 horses. Each horse is represented by a node; with individual node size associated with *gc*_*j*_, whilst the two different node colors represent *a*_*j*_ between Swiss Franches-Montagnes (FM) (green) and Warmblood (red). Top 41 key contributors are represented by an increased node size. The thickness of edges varies in proportion to the genetic distance to visualize individual relationships within the population. The five non-genotyped ancestors are indicated by an arrow.
(PDF)

S3 Fig Reordered distance matrix according to the network based population structure in cattle. From this organized heat map one can infer the shape of the identified substructure, with red and blue indicating large and small distances between the bulls.
(PDF)

S4 Fig Switch error rates of the selected subsets. Switch error rates (%) for simulated (A), horse (B) and cattle (C) for sets of 20 to 80 informative individuals, when different strategies were used to select the individuals to be included in the reference population.
(PDF)

S1 Table Population information of the top seven key contributors within sheep. Pedigree (Sire and Dam), Number of progeny (**N**_**P**_), genetic contribution score (*gc*_*j*_) and individual level of admixture (*a*_*j*_) with Awassi.
(DOCX)
