# Supplementary figures and images for: Identification of key contributors in complex population structures
Source: PLoS One. 2017 May 16;12(5):e0177638. doi: 10.1371/journal.pone.0177638 (PMC5433729; doi:10.1371/journal.pone.0177638)

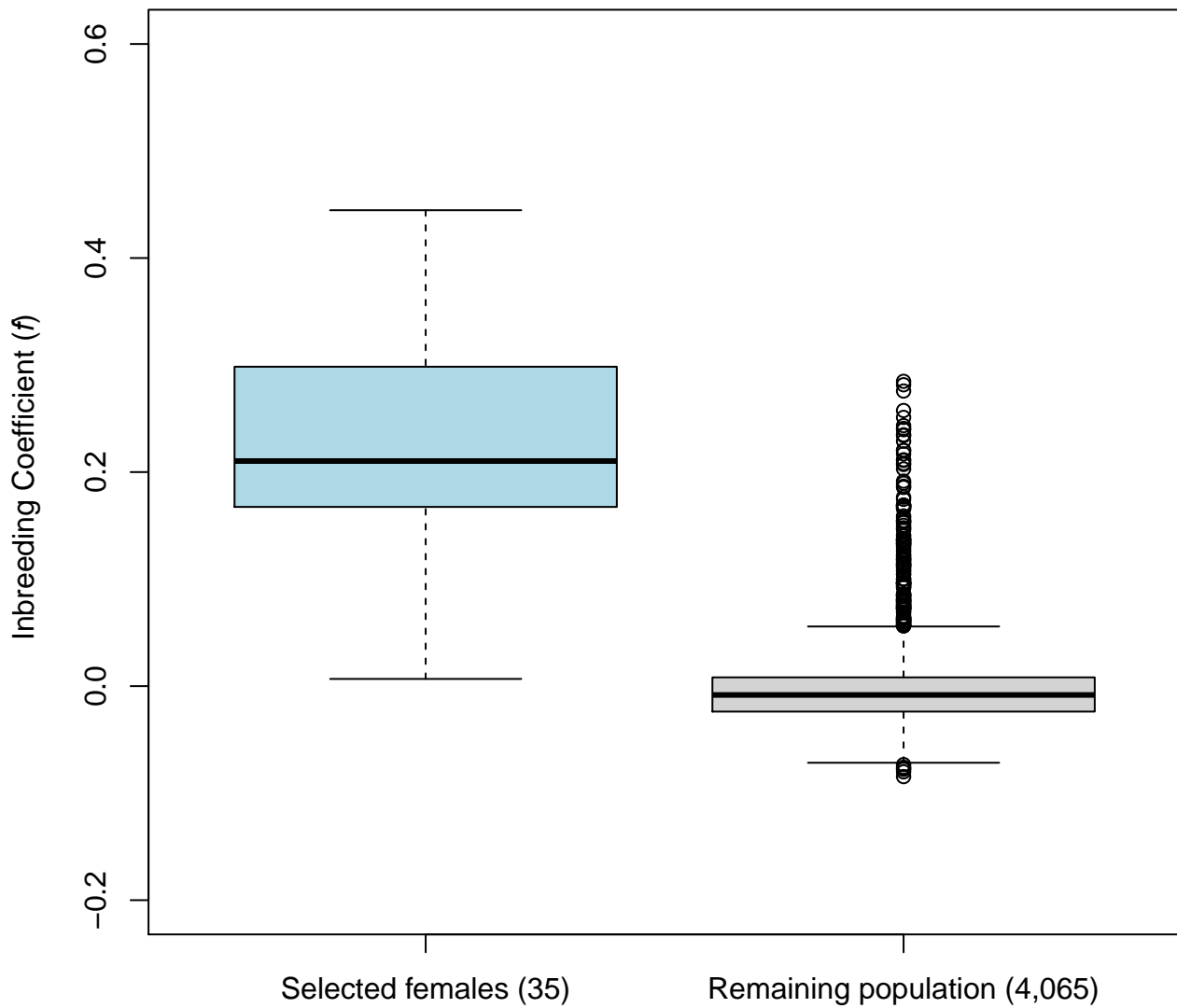

Supplement: S1 Fig — Boxplots, which indicate the median value, 25% and 75% quartiles of the inbreeding coefficient of the 35 selected females (blue) and the remaining population (grey). (PDF) [file pone.0177638.s002.pdf]

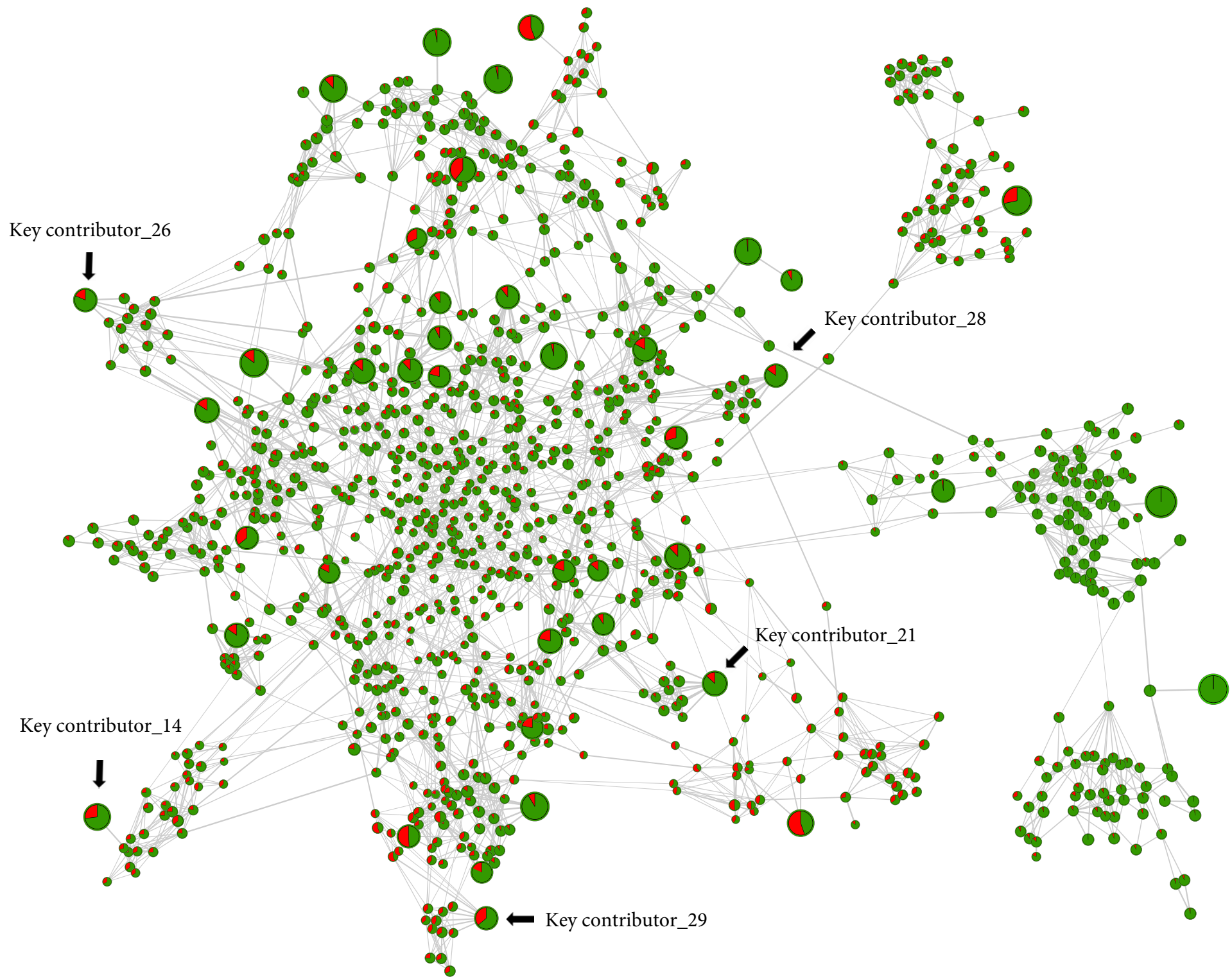

Supplement: S2 Fig — Network visualization of 1,082 horses. Each horse is represented by a node; with individual node size associated with gcj, whilst the two different node colors represent aj between Swiss Franches-Montagnes (FM) (green) and Warmblood (red). Top 41 key contributors are represented by an increased node size. The thickness of edges varies in proportion to the genetic distance to visualize individual relationships within the population. The five non-genotyped ancestors are indicated by an arrow. (PDF) [file pone.0177638.s003.pdf]

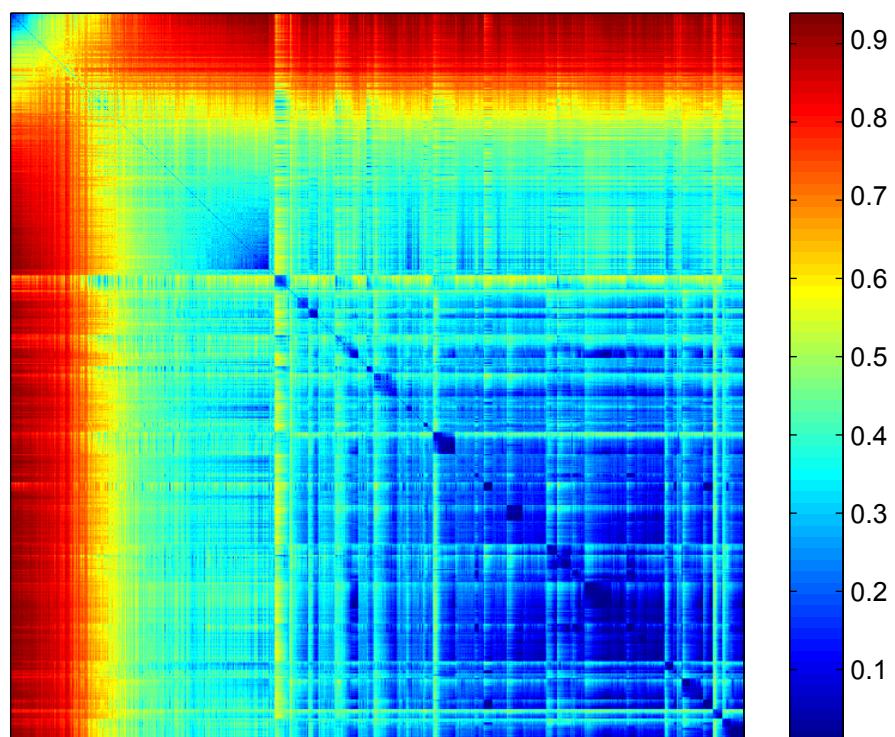

Supplement: S3 Fig — From this organized heat map one can infer the shape of the identified substructure, with red and blue indicating large and small distances between the bulls. (PDF) [file pone.0177638.s004.pdf]

**A****Simulated**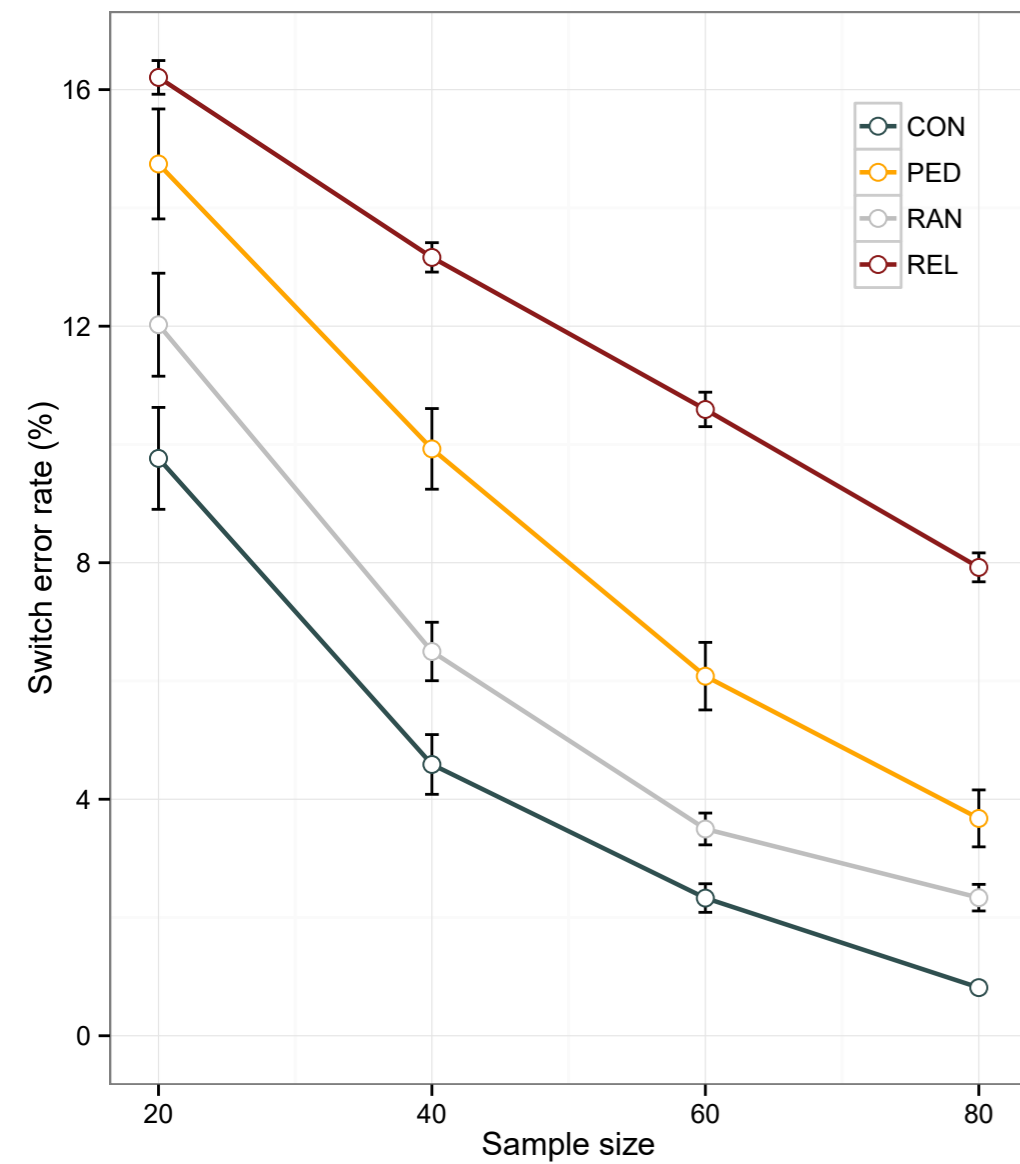**B****Horse**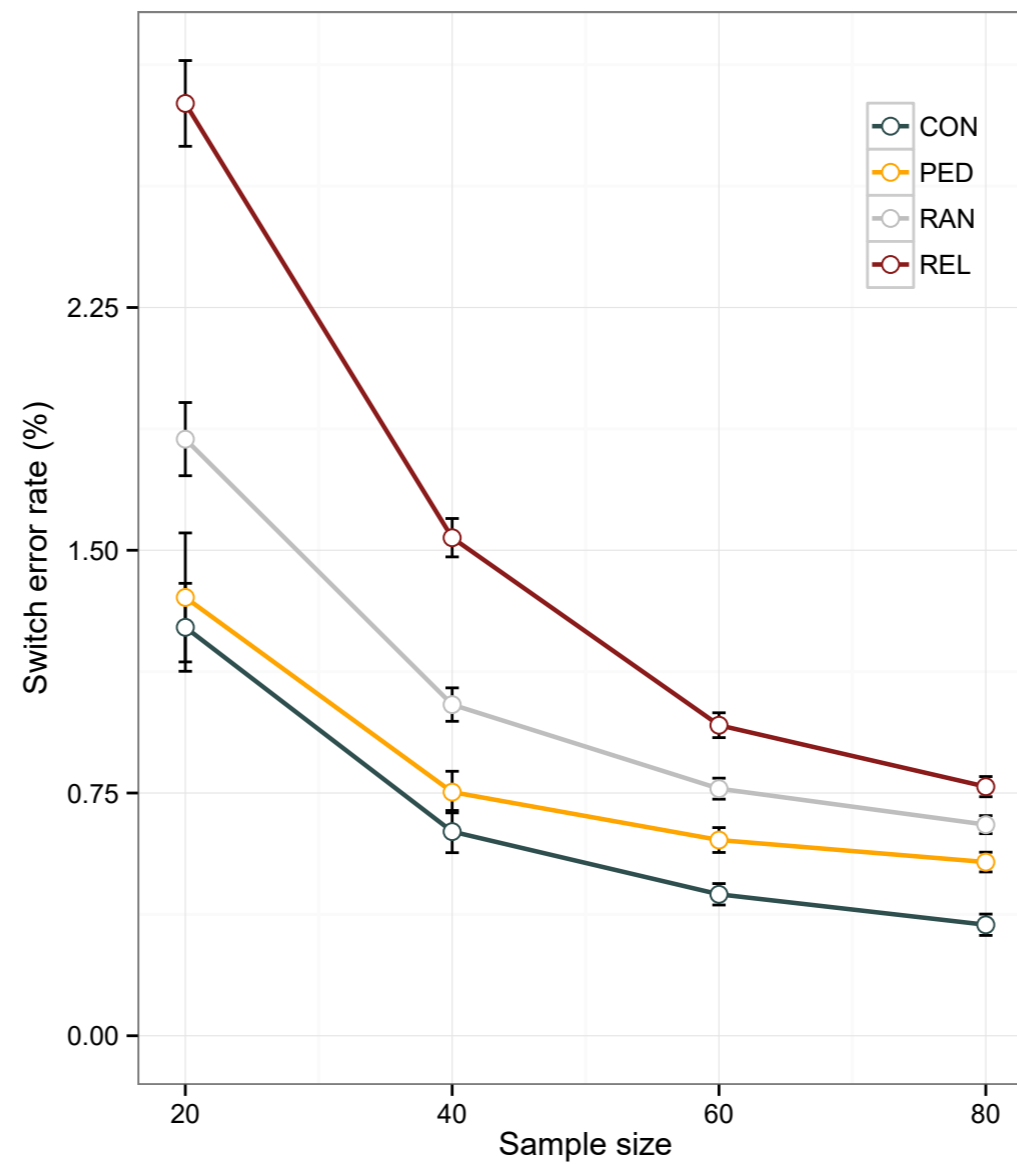**C****Cattle**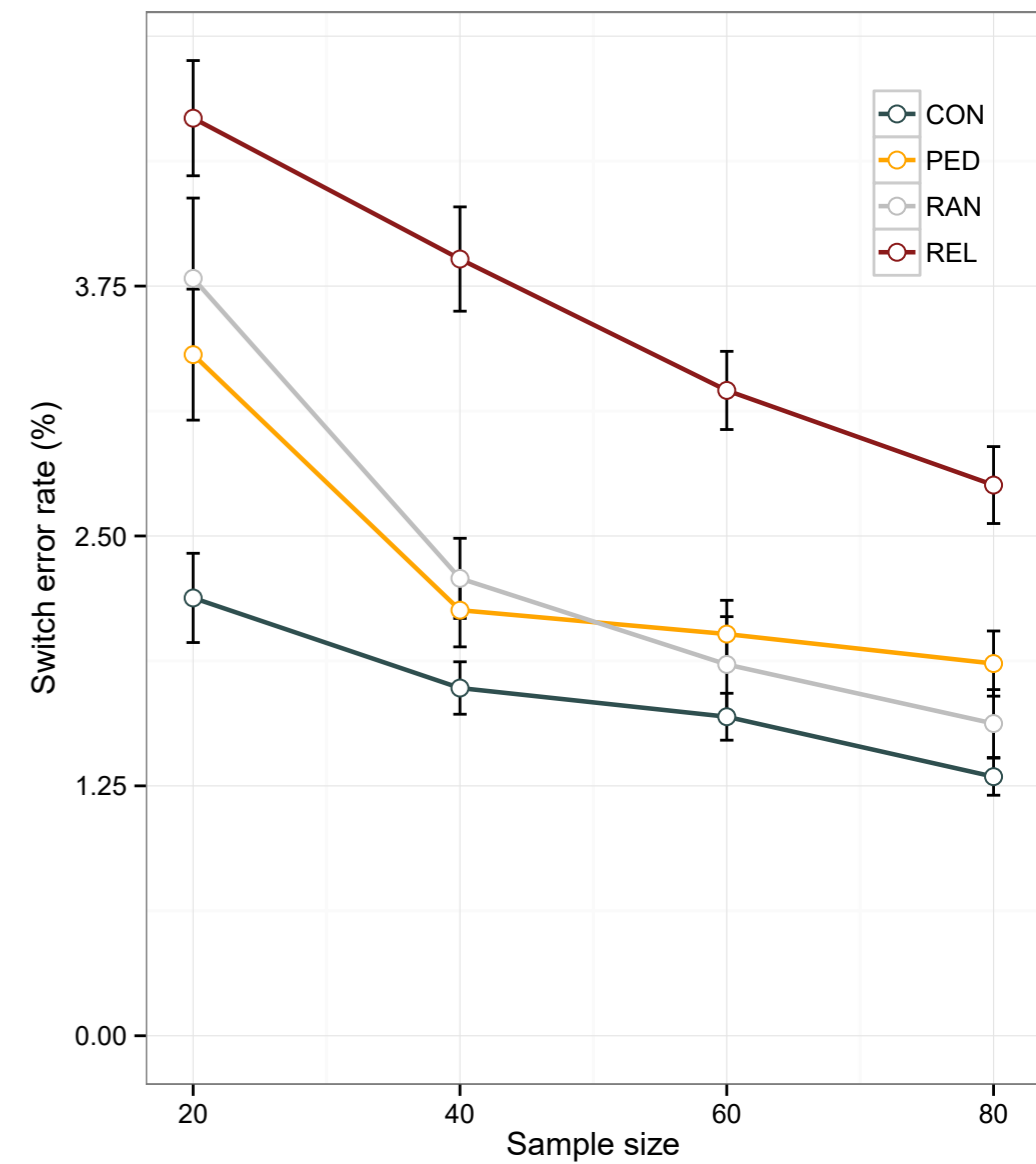

Supplement: S4 Fig — Switch error rates (%) for simulated (A), horse (B) and cattle (C) for sets of 20 to 80 informative individuals, when different strategies were used to select the individuals to be included in the reference population. (PDF) [file pone.0177638.s005.pdf]
