# Supplementary material for: Identification of key contributors in complex population structures
Source: PLoS One. 2017 May 16;12(5):e0177638. doi: 10.1371/journal.pone.0177638 (PMC5433729; doi:10.1371/journal.pone.0177638)
Supplement: S1 Table — Pedigree (Sire and Dam), Number of progeny (NP), genetic contribution score (gcj) and individual level of admixture (aj) with Awassi. (DOCX) [file pone.0177638.s006.docx]

**Supplementary Table 1.** Pedigree information, number of progeny ($\mathbf{N}_{\mathbf{P}}$), genetic contri-bution score ($\mathbf{gc}_{\mathbf{j}}$) and individual level of admixture ($\mathbf{a}_{\mathbf{j}}$) with Awassi for top seven key contributors within sheep.

| **Rank** | **Name** | **Sire** | **Dam** | **Damsire** | $\mathbf{N}_{\mathbf{P}}$ | $\mathbf{gc}_{\mathbf{j}}$ | $\mathbf{a}_{\mathbf{j}}$ |
| --- | --- | --- | --- | --- | --- | --- | --- |
| 1 | F2_Sire 1 | F1_Sire 1 | F2_Dam 1 | F1_Sire 1 | 89 | 0.628 | 0.583 |
| 2 | F2_Sire 2 | F1_Sire 2 | F2_Dam 2 | F1_Sire 2 | 67 | 0.620 | 0.352 |
| 3 | F1_Sire 2 | F0_Awassi | F0_Merino* | n.a. | 313 | 0.494 | 0.518 |
| 4 | F1_Sire 4 | F0_Awassi | F0_Merino* | n.a. | 279 | 0.493 | 0.617 |
| 5 | F1_Sire 1 | F0_Awassi | F0_Merino** | n.a. | 488 | 0.483 | 0.675 |
| 6 | F2_Sire 3 | F1_Sire 4 | F2_Dam 3 | F1_Sire 3 | 48 | 0.482 | 0.484 |
| 7 | F1_Sire 3 | F0_Awassi | F0_Merino* | n.a. | 126 | 0.477 | 0.587 |

*fine wool Merino; **medium wool Merino
